# Skin Lesion Segmentation from Dermoscopic Images Using Convolutional Neural Network

**DOI:** 10.3390/s20061601

**Published:** 2020-03-13

**Authors:** Kashan Zafar, Syed Omer Gilani, Asim Waris, Ali Ahmed, Mohsin Jamil, Muhammad Nasir Khan, Amer Sohail Kashif

**Affiliations:** 1 Department of Biomedical Engineering and Sciences, School of Mechanical and Manufacturing Engineering, National University of Sciences and Technology, Islamabad 44000, Pakistan; kashan293@gmail.com (K.Z.); asim.waris@smme.nust.edu.pk (A.W.); talpur54@outlook.com (A.A.); amer.kashif@smme.nust.edu.pk (A.S.K.); 2 Department of Electrical and Computer Engineering, Memorial University of Newfoundland, Newfoundland, St. John’s, NL A1C 5S7 P.O. Box 4200, Canada; mjamil@mun.ca; 3 Department of Electrical Engineering, University of Lahore, Lahore 54590, Pakistan; muhammad.nasir@ee.uol.edu.pk

**Keywords:** melanoma, dermoscopic images, convolutional neural networks, U-Net, ResNet, image inpainting, Jaccard Index, ROC curve

## Abstract

Clinical treatment of skin lesion is primarily dependent on timely detection and delimitation of lesion boundaries for accurate cancerous region localization. Prevalence of skin cancer is on the higher side, especially that of melanoma, which is aggressive in nature due to its high metastasis rate. Therefore, timely diagnosis is critical for its treatment before the onset of malignancy. To address this problem, medical imaging is used for the analysis and segmentation of lesion boundaries from dermoscopic images. Various methods have been used, ranging from visual inspection to the textural analysis of the images. However, accuracy of these methods is low for proper clinical treatment because of the sensitivity involved in surgical procedures or drug application. This presents an opportunity to develop an automated model with good accuracy so that it may be used in a clinical setting. This paper proposes an automated method for segmenting lesion boundaries that combines two architectures, the U-Net and the ResNet, collectively called Res-Unet. Moreover, we also used image inpainting for hair removal, which improved the segmentation results significantly. We trained our model on the ISIC 2017 dataset and validated it on the ISIC 2017 test set as well as the PH^2^ dataset. Our proposed model attained a Jaccard Index of 0.772 on the ISIC 2017 test set and 0.854 on the PH^2^ dataset, which are comparable results to the current available state-of-the-art techniques.

## 1. Introduction

Computer-aided technologies for the diagnostic analysis of medical images have received significant attention from the research community. These are efficiently designed and modified for the purposes of inter-alia segmentation and classification of the region of interest (ROI) [1], which in this instance involves cancerous regions. Needless to mention, the effective treatment of cancer is dependent on early detection and delimitation of lesion boundaries, particularly during its nascent stages because cancer generally has the characteristic tendency of delayed clinical onset [2]. Every year, nearly 17 million people are affected by cancer and about 9.6 million people die due to delayed diagnosis and treatment [3]. This makes cancer the leading causes of death worldwide [4]. In the case of skin cancer, it is one of the most prevalent types of the disease in both adults and children [5] and occurs or originates in the epidermal tissue. Various computer-aided techniques have been proposed for cancer boundary detection from dermoscopic images [3].

Among the different types of skin cancers, melanoma is not only the most dangerous and aggressive in nature due to its high metastasis rate, but also has the greatest prevalence [4]. Melanoma is a malignant type of skin cancer that develops through the irregular growth of pigmented skin cells called melanocytes [5]. It can develop anywhere on the epidermal layer of the skin and presumably may also affect the chest and back and propagate from the primary site of the cancer [6]. Its incidence rate has risen up to 4–6% annually and has the highest mortality rate among all types of skin cancers [4]. Early diagnosis is crucial as it increases the five-year survival rate up to 98% [7,8].

From the above details pertaining to the incidence and mortality rate associated with melanoma, timely diagnosis becomes all the more necessary for providing effective treatment to the affected. Insofar as the detection and segmentation of lesion boundaries, there are two streams of methodologies: first, traditional methods that usually resort to visual inspection by the clinician, and second, semi-automated and automated methods, which mostly involve point-based pixel intensity operations [9,10], pixel clustering methods [11,12,13,14], level set methods [15], deformable models [16], deep-learning based methods [17,18,19], et cetera.

Be that as it may, most of the methods being used today are not semi-automated because the accuracy associated therewith is generally prone to errors due to the following reasons: the inherent limitations of the methods [20], and changing character of dermoscopic images induced due to the florescence and brightness inhomogeneities [10]. For this very reason, the world has shifted toward more sophisticated methods, inter-alia, the convolutional neural networks (CNNs) [21].

In this paper, we intend to exploit the properties and model architectures based on CNN for skin lesion boundary delimitation and segmentation. In addition, we propose our own novelty within the already available techniques which greatly increases the segmentation accuracy, which is image inpainting. Image inpainting, together with other image preprocessing techniques such as morphological operations, is used to remove the hair structures contained within the dermoscopic images that otherwise handicap the architecture because of complexities present in the images.

This research examines the accuracy of the proposed technique together with the adoption of the proposed preprocessing method. We also benchmark our proposed scheme with other available methods by way of results through network accuracy, Jaccard Index, Dice score, and other performance metrics that aid us in comparison.

### 1.1. Literature Review

This section delineates and chalks-out the relevant work done on the issue of segmentation of skin lesions. It is done with an added emphasis and focus on the recent studies that have incorporated deep-learning methods for the aforementioned purpose of lesion segmentation.

At the outset, it is contended that accurate segmentation and delimitation of skin lesion boundaries can aid and assist the clinician in the detection and diagnosis process, and may later also help toward classification of the lesion type. There has been a gamut of studies done for the purposes of segmentation and classification of skin lesions, and for a general survey of these, the reader can refer the following two papers authored by Oliveira et al. [3], and Rafael et al. [22].

We hereinafter present a review of the literature vis-à-vis two aspects (i.e., preprocessing and segmentation techniques, respectively). Both aspects have a direct effect on the outcome of the results (the prediction) and therefore, both are catered into the broader scheme of methodology presented in this paper. Additionally, since dermoscopic images have varying complexities and contain different textural, intensity, and feature inhomogeneity, it becomes necessary to apply prior preprocessing techniques so that inhomogeneous sections can be smoothened out.

#### 1.1.1. Preprocessing Techniques

Researchers encounter complications while segmenting skin lesions due to low brightness and the noise present in the images. These artifacts affect the accuracy of segmentation. For better results, Celebi et al. [23] proposed a technique that enhances image contrast by searching for idyllic weights for converting RGB images into grayscale by maximizing Otsu’s histogram bimodality measure. Optimization resulted in a better adaptive ability to distinguish between tumor and skin and allowed for accurate resolution of the regions, whereas Beuren et al. [24] described the morphological operation that can be applied on the image for contrast enhancement. The lesion is highlighted through the color morphological filter and simply segmented through binarization. Lee et al. [25] proposed a method to remove hair artifacts from dermoscopic images. An algorithm based on morphological operations was designed to remove hair like artifacts from skin images. Removing hair, characterized as noise, from skin images has a noteworthy effect on segmentation results. A median filter was found to be effective on noisy images. A nonlinear filter was applied to images to smooth them [26]. Celebi et al. [27] established a concept where the size of the filter to be applied must be proportional to the size of the image for effective smoothing.

Image inpainting is a preprocessing technique used for both removing parts from an image and for restoration purposes, so that the missing and damaged information in images is restored. It is of vital importance in the field of medical imaging and through its application, unnecessary structures or artifacts from the images (i.e., hair artifacts in skin lesions images) can be removed [28,29,30].

#### 1.1.2. Segmentation Techniques

Most image segmentation tasks use traditional machine learning processes for feature extraction. The literature explains some of the important techniques used for accurate segmentation. Jaisakthi et al. [31] summarizes a semi-supervised method for segmenting skin lesions. Grab-cut techniques and K-means clustering are employed conjunctively for segmentation. After the former segments the melanoma through graph cuts, the latter fine-tunes the boundaries of the lesion. Preprocessing techniques such as image normalization and noise removal measures are used on the input images before feeding them to the pixel classifier. Mohanad Aljanabi et al. [32] proposed an artificial bee colony (ABC) method to segment skin lesions. Utilizing fewer parameters, the model is a swarm-based scheme involving preprocessing of the digital images, followed by determining the optimum threshold value of the melanoma through which the lesion is segmented, as done by Otsu thresholding. High specificity and Jaccard Index are achieved by this algorithm.

Pennisi et al. [33] introduced a technique that segments images using the Delaunay triangulation method (DTM). The approach involves parallel segmentation techniques that generate two varying images that are then merged to obtain the final lesion mask. Artifacts are removed from the images after which one process filters out the skin from the images to provide a binary mask of the lesion, and similarly, the other technique utilizes Delaunay triangulation to produce the mask. Both of these are combined to obtain the extracted lesion. The DTM technique is automated and does not require a training process, which is why it is faster than other methods. M Emre Celebi et al. [34] provides a brief overview of the border detection techniques (i.e., edge based, region based, histogram thresholding, active contours and clustering, etc.) and especially pays attention to evaluation aspects and computational issues. Lei Bi et al. [35] suggested a new automated method that performed image segmentation using image-wise supervised learning (ISL) and multiscale super pixel based cellular automata (MSCA). The authors used probabilistic mapping for automatic seed selection that removes user-defined seed selection; afterward, the MSCA model was employed for segmenting skin lesions. Ashnil Kumar et al. [36] introduced a fully convolutional network (FCN) based method for segmenting dermoscopic images. Image features were learned from embedded multi-stages of the FCN and achieved an improved segmentation accuracy (than previous works) of skin lesion without employing any preprocessing part (i.e., hair removal, contrast improvement, etc.). Yading Yuan et al. [37] proposed a convolution deconvolutional neural network (CDNN) to automate the process of the segmentation of skin lesions. This paper focused on training strategies that makes the model more efficient, as opposed to the use of various pre- and post-processing techniques. The model generates probability maps where the elements correspond to the probability of pixels belonging to the melanoma. Berseth et al. [38] developed a U-Net architecture for segmenting skin lesions based on the probability map of the image dimension where the ten-fold cross validation technique was used for training the model. Mishra [17] presented a deep learning technique for extracting the lesion region from dermoscopic images.

This paper combines Otsu’s thresholding and CNN for better results. U-Net based architecture was used to extract more complex features. Chengyao Qian et al. [39] proposed an encoder decoder architecture for segmentation inspired by DeepLab [40] and ResNet 101 was adapted for feature extraction. Frederico Guth et al. [41] introduced a U-Net 34 architecture that merged insights from U-Net and ResNet. The optimized learning rate was used for fine tuning the network and the slanted triangular learning rate strategy (STLR) was employed.

## 2. Materials and Methods

### 2.1. Dataset Modalities

**Figure 1:**
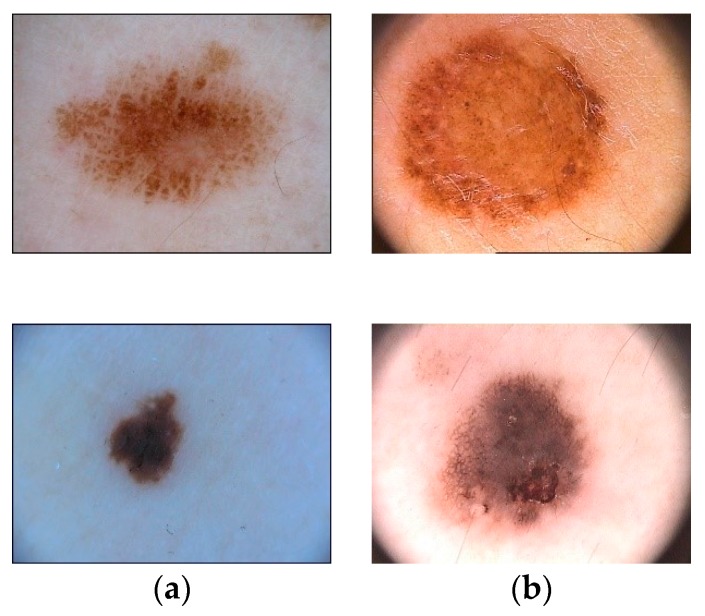
Examples of the ISIC-17 dataset (**a**) and PH^2^ dataset (**b**).

We trained and tested our CNN model on dermoscopic skin images acquired from two publicly accessible datasets (i.e., PH^2^ [42] and ISIC 2017 [43]), the latter provided by the “International Skin Imaging Collaboration” (ISIC). Example images from both datasets are shown in Figure 1.

We compared our model in the task of Lesion Segmentation, part 1 of the 2017 ISBI Skin Lesion Analysis Toward Melanoma Detection challenge. We evaluated our model on the ISIC-17 test data consisting of 600 images to compare its performance with state-of-the-art pipelines. Additionally, our model was also tested on the PH^2^ dataset with its 200 dermoscopic images including 40 melanoma, 80 common nevi, and 80 atypical nevi images.

### 2.2. Proposed Methodology

In this section, we introduce our devised methodology, which was trained and tested on the datasets (details presented later), and the subsequent results are reported and discussed. At the outset, it is pertinent to mention that we proposed a method that out-performed other similar available methods, both in terms of model accuracy and in pixel-by-pixel similarity measure, also called the intersection over union overlap (sometimes also referred to as the Jaccard Index). We herein proceed to describe, point by point, the various subsections of the proposed method.

#### 2.2.1. Image Preprocessing

Images are preprocessed using resizing, scaling, hair removal and data centering techniques before being given as input to the CNN model. For noise removal, morphological operations are applied. We obtained promising results by applying preprocessing practices, which are as follows.

Image Resizing: It is good practice to resize images before they are fed into the neural network. It allows the model to convolve faster, thereby saving computational power and dealing with memory constraints. Dermoscopic images vary in size and to overcome such individual differences, the images and their corresponding ground truths are down sampled to 256 × 256 resolution. All the RBG images are in the JPEG file format while the respective labels are in the PNG format.

Image Normalization and Standardization: Images are normalized before training to remove poor contrast issues. Normalization changes the range of pixel values, rescaling the image between 0 and 1 so that the input data is centered around zero in all dimensions. Normalization is obtained by subtracting the image from its mean value, which is then divided by the standard deviation of the image.

**Figure 2:**
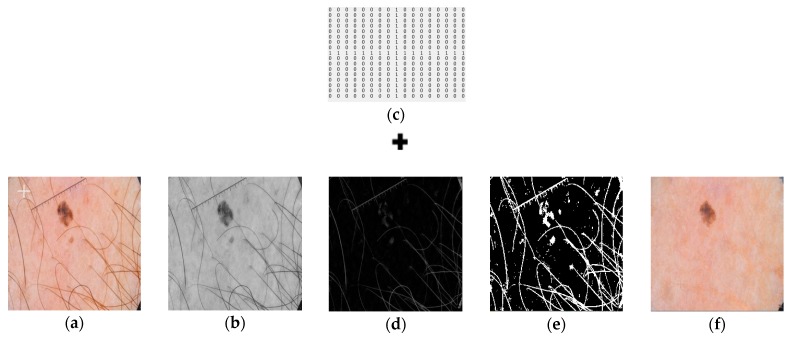
Representation of a single image as it is passed through the hair removal algorithm. Left to right: (**a**) Input image; (**b**) Grayscale image; (**c**) Cross shaped structuring element employed during morphological operations; (**d**) Image obtained after applying black top-hat filter; (**e**) Image after thresholding; (**f**). Final image obtained as output.

Hair Removal: Dermoscopic images contain hair-like artifacts that cause issues while segmenting lesion regions. A series of morphological operations are applied to the image to remove these hair-like structures. The inpainting algorithm [44] is then applied to replace the pixel values with the neighboring pixels, explained as follows:

- Conversion of RGB (Figure 2a) to grayscale image (Figure 2b);
- Black top-hat filter [45,46] is applied to the grayscale image;
- Inpainting algorithm is implemented on the generated binary mask; and
- Inpainting of the hair occupied regions with neighboring pixels.

A 17 × 17 cross shaped structuring element is defined, as shown in Figure 2c. Black top-hat (or black hat filter) filtering is obtained by subtracting closing of image from original image. If A is the original input image and B is the closing of the input image, then black top-hat filter is defined by Equation (1):

Black Hat(A) = A_BH_ = (A · B) − A (1)

Closing morphological operation is the erosion of the dilation of set A and B. Closing fills small holes in the region while keeping the initial region sizes intact. It preserves the background pixels that are like the structuring element, while eliminating all other regions of the background.

The image obtained after applying the closing operation on a grayscale image is subtracted from the image itself to obtain hair like structures. Binary mask of the hair elements is obtained by applying a threshold value of “10” on the image obtained from the black top-hat filter. Images obtained from the black top-hat filter and after thresholding, respectively, are highlighted in Figure 2d,e.

The image based on the fast marching method was employed [47]. The inpainting algorithm replaces the hair structures with the bordering pixels of the image to restore the original image. This technique is commonly used in recovering old or noisy images. The image to be inpainted and the mask obtained after thresholding was used to inpaint those hairy regions that were extracted with the neighboring pixels and output was achieved (Figure 2f).

#### 2.2.2. Model Architecture

Deep learning architectures are currently being used to solve visual recognition and object detection problems. CNN models have shown good impact over semi-automated methods for semantic segmentation. The U-Net architecture, which is based on an encoder–decoder approach, has revealed significant results in medical image segmentation. The output of these networks are binary segmentation masks.

In general, CNN models are the combination of layers (i.e., convolutional, max pooling, batch normalization, and activation layer). CNN architectures have been widely used in computer assisted medical diagnostics.

**Figure 3:**
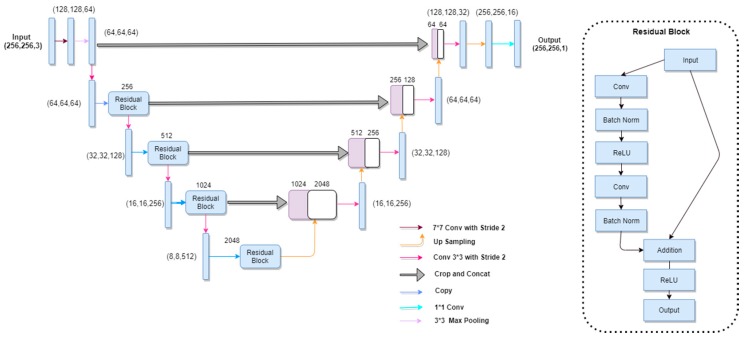
Schematic diagram representing UResNet-50. The encoder shown on the left is the ResNet-50, while the U-Net decoder is shown on the right. Given in the parenthesis is the channel dimensions of the incoming feature maps to each block. Arrows are defined in the legend.

For this purpose, a CNN architecture was trained on an ISIC 2017 dataset. The network architecture (as shown in Figure 3) takes insight from both U-Net and ResNet. The contracting path (convolutional side) is based on the ResNet architecture, and the expansive path (deconvolutional side) is based on the U-Net pipeline. Overall, the network performs in an encoder–decoder fashion and is composed of 50 layers (ResNet-50). Input images of resolution 256 × 256 are fed into the model. The convolutional network architecture is shown in Table 1.

On the contracting side, after the first convolutional layer, a max pooling layer is defined with a kernel of 3 × 3 and a stride of 2 that halves the input dimension. Repetitive blocks are introduced with three convolutional layer per block; the 1 × 1 convolutional layer is defined before and after each 3 × 3 convolutional layer. It reduces the number of channels in the input before the 3 × 3 convolutional layer and again, the 1 × 1 is defined to restore dimensions. This is called the “Bottleneck” design, which reduces the training time of the network.

After 5 units of downsampling, the dimension ranges to 8 × 8 and 2048 filters. In contrast, the deconvolutional side or expansive path (as shown in Table 2) consists of 10 layers that perform deconvolution.

#### 2.2.3. Network Training

We trained our model for 100 epochs and applied data augmentation during runtime, which enhances the performance as more data increases the predictability of the model so that it can classify better, thereby producing a significant effect on the segmentation results. We rotated images in three dimensions, which increased the dataset thricefold.

Early stopping is defined and the learning rate is reduced if the model loss does not decrease for 10 epochs. Our model stopped after approximately 70 epochs. Transfer learning was employed for training the model on our dataset, utilizing pre-trained weights obtained through training on the ImageNet dataset. Table 3 shows the hyperparameters used to train our model.

## 3. Results

### Model Evaluation

**Figure 4:**
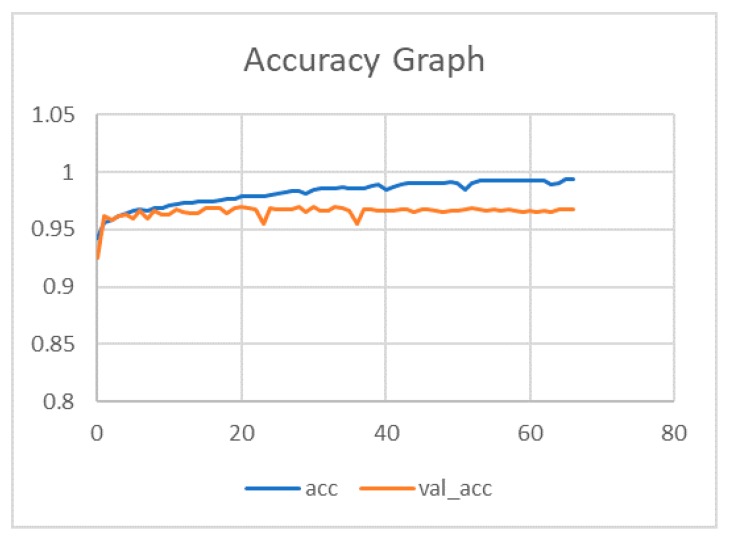
Training and validation accuracy of the proposed convolutional neural network model for 70 epochs.

Our model was evaluated on images obtained from the International Skin Imaging Collaboration ISIC 2017. We trained our CNN model on the training group of ISIC 2017, which consisted of 2000 skin lesion images. During this process, a total training accuracy of 0.995 was obtained for 70 epochs. The variations of accuracy between the training and validation group during training is highlighted in Figure 4.

**Figure 5:**
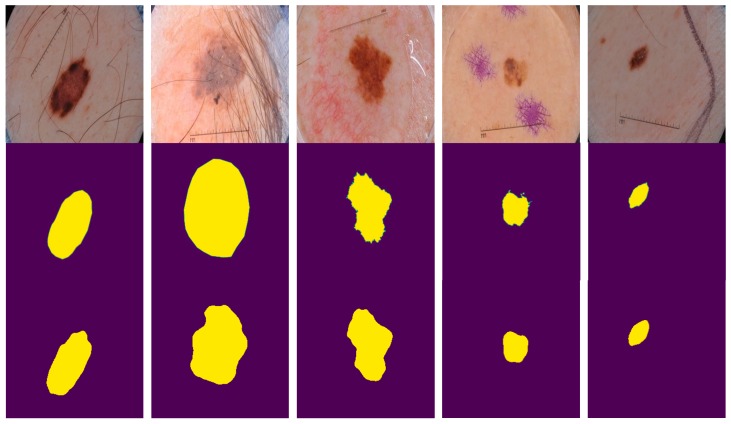
Example results of multiple patients. The first row contains the original images of five patients from the test set. The second row contains corresponding ground truths as provided. The third row contains predicted masks from the proposed method.

The model was tested on the validation and test set taken from the ISIC 2017 dataset. Furthermore, the model was also tested on the PH^2^ dataset comprising of 200 dermoscopic images. The ground truths were also available in order to check the performance of the proposed CNN model. All images went through the preprocessing step before being fed into the CNN architecture as described earlier. Parameters of convolutional layers were set during the training process. During the evaluation process, the model parameters were not changed in order to assess our model’s performance on the pre-set parameters. The results of multiple subjects are shown in Figure 5.

**Figure 6:**
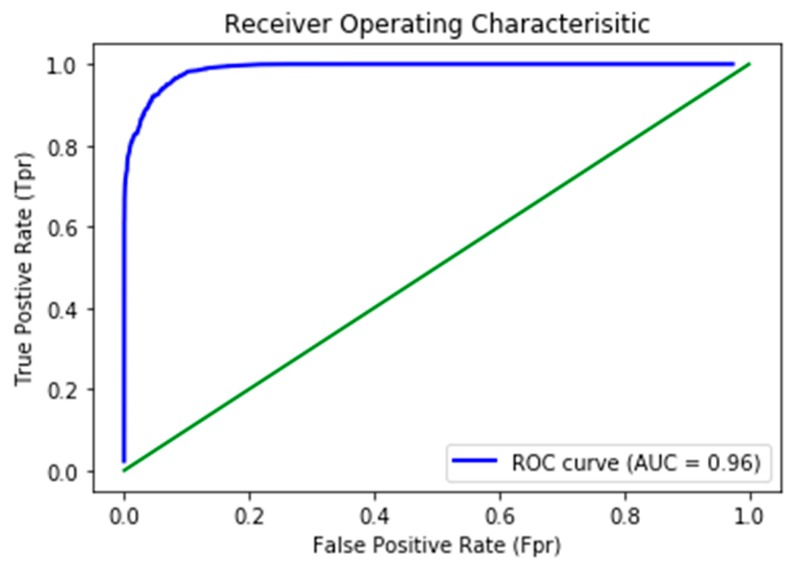
Receiver operative characteristics (ROC) curve generated on the ISIC-17 test set.

The receiver operative characteristics (ROC) curve was used to evaluate the performance binary classifiers. ROC is a plot between the true positive rate (Sensitivity) as a function of false positive rate (Specificity) at different thresholds. This study emphasizes segmenting the lesion region, with 1 representing the lesion region and 0 representing the black region of the image. The ROC curve is the best evaluation technique that defines separability between classes. Each datapoint in a curve shows the values at a specific threshold. Figure 6 shows the ROC curve of the model on the ISIC test set.

The ROC curve dictates the model’s capability to distinguish between classes accurately. The higher the area under the curve, the higher the network’s ability to distinguish two classes more precisely (i.e., either 0 or 1). The AUC of our proposed model was 0.963, illustrating the model’s remarkable competence of differentiability.

## 4. Benchmarks

### 4.1. Comparison with Different Frameworks

The model was tested both with and without preprocessing on the ISIC-17 dataset to ascertain the efficiency of the hair removing algorithm. A Jaccard of 0.763 (as shown in Table 4) was achieved when the inpainting algorithm was not employed to remove the hair structures from the images, which improved considerably to 0.772 with the implementation of the preprocessing technique.

For evaluation, we compared our results with the existing deep learning frameworks (enlisted in Table 5) that had been tested on the ISIC-17 dataset. FCN-8s [48] achieved a JI (0.696) and DC (0.783), respectively. Although our proposed method was the deepest among the below listed frameworks, we improved the results by balanced data augmentation and reduced overfitting. Simple U-Net obtained a JI of 0.651 and a DC of 0.768.

Our proposed method is a combination of the ResNet50 based encoder and U-Net based decoder, which achieved a Jaccard index of 0.772 and Dice coefficient of 0.858.

### 4.2. Comparison with Top 5 Challenge Participants of Leaderboard

The intent was that this research would segment the lesion regions with higher accuracy when compared to other methods. Three different group of images were used to validate our network: (1) the ISIC 2017 test group; (2) ISIC 2017 validation group; and the (3) PH^2^ dataset. The test group consisted of 600 dermoscopic images and the validation group was composed of 150 images. The PH^2^ dataset is a renowned dataset and was used for further evaluation of our network and benchmarking our results with existing methods and participants in the challenge. Table 6 depicts our results in terms of the Jaccard Index as per the challenge’s demand, in comparison with the top five participants from the ISIC-17 Challenge. The top ranked participant Yading Yaun et al. [21] obtained a Jaccard index of 0.765.

Different methods has been employed for segmentation purpose. The second ranked participant Matt Berseth et al. [51] obtained a JI (0.762) by employing a U-Net framework. Our technique achieved a Jaccard index of 0.772 with the proposed technique stated earlier. Based on our results, our model performed better than the existing techniques used in the associative field of study.

### 4.3. Evaluation of Model on the PH^2^ Dataset

To evaluate the robustness of our proposed model, we further tested the architecture on the PH^2^ dataset and compared our segmentations with the existing state-of-the-art techniques. The results are listed below in Table 7. Our method achieved promising results. FCN-16s achieved a JI of 0.802 and DC of 0.881, respectively. Another framework, Mask-RCNN attained a JI of 0.839 and a DC of 0.907 on the PH^2^ dataset.

## 5. Conclusions

Skin lesion segmentation is a vital step in developing a computer aided diagnosis system for skin cancer. In this paper, we successfully developed a skin lesion segmentation algorithm using CNN with an advanced hair-removal algorithm that effectively removed hair structures from the dermoscopic images, improving the accuracy considerably. We tested our model architecture on the ISIC-2017 dataset and PH^2^ dataset, and the Jaccard index obtained thereof was 0.772 and 0.854, respectively. Our proposed method achieved promising results compared with the state-of-the-art techniques in terms of the Jaccard index. Furthermore, our CNN model was tested on a PH^2^ dataset along with the ISIC-17 test set and produced better segmentation and performed better than the existing methods in the literature. Empirical results show that the combination of the U-Net and ResNet shows impressive results.

The limited training data used requires extensive augmentation to prevent the model from overfitting. A large dataset is therefore needed for better accuracy and generalization of the model. Furthermore, for it to achieve state-of-the-art results, the model was made to be complex and efficient, which takes more time to train as opposed to the conventional U-Net.

Our future work includes using a larger dataset to reduce overfitting problems and hyper tuning the parameters for more effective training. Additionally, a conditional random field (CRF) application can also be applied to refine the model output.

## Figures and Tables

**Table 1:** Convolutional network architecture based on ResNet-50.

Layer Name	Output Size	Kernel Size & No. of Filters
*Conv 1*	128 × 128	7 × 7, 64, Stride 2
*Max Pooling*	64 × 64	3 × 3, Stride 2
*Conv 2*	64 × 64	$\left[\begin{array}{c} 1\times 1\times 64\\ 3\times 3\times 64\\ 1\times 1\times 256 \end{array}\right]\;$× 3
*Conv 3*	32 × 32	$\left[\begin{array}{c} 1\times 1\times 128\\ 3\times 3\times 128\\ 1\times 1\times 512 \end{array}\right]\;$× 4
*Conv 4*	16 × 16	$\left[\begin{array}{c} 1\times 1\times 256\\ 3\times 3\times 256\\ 1\times 1\times 1024 \end{array}\right]\;$× 6
*Conv 5*	8 × 8	$\left[\begin{array}{c} 1\times 1\times 512\\ 3\times 3\times 512\\ 1\times 1\times 2048 \end{array}\right]\;$× 3

**Table 2:** The deconvolution architecture based on U-Net.

Layer Name	Kernel	Output Size & No. of Filters
U1	2 × 2	16 × 16 × 2048
D1	3 × 3	16 × 16 × 256
D2	3 × 3	16 × 16 × 256
U2	2 × 2	32 × 32 × 256
D3	3 × 3	32 × 32 × 128
D4	3 × 3	32 × 32 × 128
U3	2 × 2	64 × 64 × 128
D5	3 × 3	64 × 64 × 64
D6	3 × 3	64 × 64 × 64
U4	2 × 2	128 × 128 × 64
D7	3 × 3	128 × 128 × 32
D8	3 × 3	128 × 128 × 32
U5	2 × 2	256 × 256 × 32
D9	3 × 3	256 × 256 × 16
D10	3 × 3	256 × 256 × 16
Output	1 × 1	256 × 256 × 1

**Table 3:** Hyperparameters maintained during training.

Name	Value
Input Size	256 × 256 × 3
Batch Size	16
Learning Rate	1 × 10^−3^
Optimizer	Adam
Epoch	100
Loss Function	Binary Crossentropy

**Table 4:** Model performance on the ISIC-2017 test set.

Methods	Jaccard Index
No Preprocessing	Preprocessed
0.763	0.772

**Table 5:** Comparison with different frameworks on the ISIC-2017 test set.

Methods	Jaccard Index	Dice Coefficient
FCN-8s [47]	0.696	0.783
U-Net [48]	0.651	0.768
II-FCN [49]	0.699	0.794
Auto-ED [50]	0.738	0.824
FCRN [18]	0.753	0.839
Res-Unet (Proposed)	0.772	0.858

**Table 6:** Comparison of results with the challenge participants.

Authors	Model	Jaccard Index
Yading Yuan et al. [21]	CDNN	0.765
Matt Berseth et al. [51]	U-Net	0.762
Popleyi et al. [52]	FCN	0.760
Euijoon Ahn et al. [35]	ResNet	0.758
Afonso Menegola et al. [53]	VGG16	0.754
Proposed Method	Res-Unet	0.772

**Table 7:** Comparison with different frameworks on the PH^2^ Dataset

Methods	Jaccard Index	Dice Coefficient
FCN-16s	0.802	0.881
DeeplabV3+	0.814	0.890
Mask-RCNN	0.830	0.904
Multi-Stage FCN	-	0.906
SSLS	-	0.916
Res-Unet (Proposed)	0.854	0.924
